# Building Better Public Health Policy Knowledge: The Case for Pluralism

**DOI:** 10.34172/ijhpm.9659

**Published:** 2026-03-31

**Authors:** Patrick Harris

**Affiliations:** ^1^International Centre for Future Health Systems, University of New South Wales, Sydney, NSW, Australia.; ^2^Centre for Health Equity Training, Research and Evaluation, Population Health and Epidemiology Unit, South Western Sydney Local Health District, Liverpool, NSW, Australia.

 Given the multiple challenges facing public health across the globe it is timely to reflect on how different types of knowledge are of use for public health policy. Public health policy, like all public policy, operates in complex, contested environments where multiple forms of knowledge are needed—both *for *policy (technical evidence to inform decisions and implementation) and *about* policy (socio-political to understand systems, processes, and power).^1,2^ This viewpoint reflects on how four foundational approaches^[1]^—positivism, pragmatism, critical social science (CSS), and critical realism (CR)—offer distinct yet complementary contributions to public health policy knowledge generation. I use climate change and populism—along with health equity, my professional focus—as concrete examples. My purpose is to motivate a reflective lens about the value and complementary use of different approaches for effectively generating, commissioning, reviewing, or using evidence. I proceed in three steps. First, I introduce climate change and populism as illustrative challenges. Second, I compare the four approaches in terms of causation, methods, macro-micro orientation, methods, and theory use. Third, I then detail hypothetical research protocols for the first three and suggest CR as a connecting meta-methodology. I conclude by reiterating the necessity of complementarity despite systematic institutional barriers.

## Climate Crisis and Public Health

 The climate emergency is among public health’s most complex policy challenges, demanding clarity about the kinds of knowledge we use to understand and act.^4^ Quantitative predictions and technical measurement of risks, exposures and outcomes are essential – for example estimating temperature related health outcomes and shifting disease patterns. Yet purely technical approaches often factor out the very social and political determinants that shape exposure and adaptive capacity – housing quality, energy affordability, labour protections, and local infrastructure – by treating these as confounders or as background context. Practical, locally tailored solutions such as weather alerts, adjusted rostering, and community cooling spaces are necessary, but they struggle to scale and influence the upstream drivers that configure local vulnerability. Systemic forces are at the root of the climate crisis – driven by growth at all costs policies and economic models, and governance structures that maintain the power of polluting industries. But such critical policy analysis can focus on critique rather than pathways to change.

## Populism and Public Health

 Rising populism challenges public health by eroding trust, amplifying misinformation, and privileging short-term political gains over long-term planning.^5,6^ Positivist studies can measure compliance and trust trends but cannot on their own explain why certain communities resist directives. Local interventions can be designed to co-create engagement and trust-building, yet risk tokenism and context-dependency if upstream determinants—poverty, precarious work, political disenfranchisement—are left unaddressed. Critical analysis of those macro determinants (media concentration, exclusionary governance, austerity) helps to confront political expediency, power, and erosion of public trust in institutions, but can be sidelined as out of touch with everyday lived realities.

## Comparing the Approaches

Table articulates, compares, and contrasts the core features of each approach. The table clarifies what kind of types of evidence each approach can—and cannot—produce.

**Table:** Comparisons of Approaches to Public Health Policy Knowledge Generation

**Feature**	**Positivism**	**Pragmatism**	**CSS**	**CR**
Ontology	Reality is objective and observable	Reality is shaped by experience and context	Social reality shaped by power, discourse, material and historical structures	Reality is stratified, generative, mind-independent; mechanisms operate at multiple levels
Epistemology	Knowledge through empirical observation and measurement	Knowledge through practical experience and usefulness	Knowledge is situated, political, historically contingent	Knowledge is fallible and theory-laden; aims to identify causal mechanisms
Theory use	Describes observable phenomena	Guides action and problem-solving	Critiques domination, inequality, ideology; foregrounds power and agency	Explains deep generative mechanisms producing observable patterns
Methods	Quantitative, experimental, statistical	Mixed methods, applied research, abduction	Qualitative, interpretive, genealogical, discourse analytic	Retroduction, multi-level analysis, explanatory mixed methods
Strengths	Strong in measurement and prediction; tight causal inference	Flexible and outcome-oriented; useful for implementation	Strong on analysing power, inequality, ideology; normative and emancipatory	Strong explanatory depth; investigates structure and agency; ontological clarity
Weaknesses	Treats context as noise and power as external to causation	Can be overly instrumental; local focus can miss structural drivers; risks reproducing inequities	May lack causal clarity; fragmented across traditions; can prioritise critique over explanation and action	Can be abstract and conceptually dense; specialised language can be rhetorically off-putting and self-limiting
Application in public health	Epidemiology, surveillance, clinical trials	Program evaluation, implementation research	Health equity, structural critiques, social determinants, intersectionality, policy power analysis	Explaining mechanisms behind inequities; multi-level causal pathways; realist evaluation foundations
View of causality	Linear and observable	Contextual and practical (causation as a tool for action)	Relational, historical, non-linear (structures condition agency and outcomes)	Generative, layered, non-linear mechanisms in open systems
Role of values	Science is value-free	Values guide what works	Values are explicit, political, and central to critique and emancipation	Values shape inquiry; explanatory critique provides a warrant for change

Abbreviations: CSS, critical social science; CR, critical realism.

 I now summarize the core stance, views on causation, macro/micro implications, and use of theory for each.

 Positivism preferences measurement of factual observations and direct causal inference using experimental, quasi-experimental, and statistical designs.^7^ Strengths include precision in estimating effects; limitations include treating—and factoring out of the analysis—context as noise and power as external to causation.^8^ Causation is operationalised as regularities between variables, pushing attention toward micro-level or program specific causal relationships (eg, rising temperatures causing more heat-related admissions; populist governance reducing vaccine uptake). Positivism does extend to macro analysis via large-scale surveillance and predictive modelling.^7^ The use of theory is “a priori” for *deductive* hypothesis testing. Social epidemiologists, specifically those concerned with health equity, are recently debating how to reconcile variable-based inference with multilevel, interdependent causation and feedback loops rooted in societal conditions, with calls for reflexivity about upstream social and political conditions and mechanisms.^8,9^

 Pragmatism is concerned with what works in specific contexts, emphasising local knowledge, predetermined practical outcomes, and real-world feasibility.^10^ Pragmatism rejects both positivist efforts to reduce complexity to controllable variables and CSS/CR tendencies to explain the world through structures that sit beyond lived experience.^10^ Causation is a tool for problem solving in lived contexts^11^; pragmatists commonly use mixed methods, co-design, participatory approaches, and implementation science. That preference delivers highly responsive local research [and or] evaluation, but limits the ability to generalize, explain, or achieve lasting impact when macro-level forces produce the very conditions that local interventions confront. In some cases, local optimisation can unintentionally reproduce inequities by adapting to unjust conditions rather than transforming them. Pragmatists treat theory instrumentally as useful only for explaining lived experience,^10^ and prioritise *inductive* analysis grounded in the data, with theory playing a supporting, late arriving, role. Recent work invites *abductive* analysis^12^ to introduce theory for inferential purposes while retaining inquiry anchored in lived experience.

 CSS focuses on macro-level structures, institutions, and ideologies—capitalism, racism, patriarchy, coloniality—that produce and reproduce [health] inequities.^3^ CSS is normative and emancipatory, beginning from political and ethical commitments to justice. CSS understands causation as relational, historical, and non-linear: outcomes emerge from the interplay of structural constraint or enablement, path dependence, power relations, and lived practices.^13^ CSS is strongest at macro-level diagnosis yet can be open to criticism for not translating structural critique into concrete change focused practical action.^14^ Theory is central and explicit. CSS draws on grand and middle-range theories (eg, Marxism, feminism, postcolonial, and critical race theory) to frame inquiry and interpretation. Crucially, these theoretical positions do not always align and can be critical of each other.^15^ Methods are qualitative, interpretive, and reflexive; validity includes theoretical adequacy in addition to observation.

 CR is a meta-methodology concerned with explaining how structures, practices, and events interact in stratified social systems, where structure (macro, meso) and agency (meso, micro) are separate but interacting.^16^ Like CSS CR is emancipatory but its normativity is expressed through explanatory critique rooted in causal analysis: if a harmful outcome can be shown to arise from identifiable causal mechanisms, then there is a *rational obligation* to change those mechanisms. CR sees reality as stratified and layered (real/actual/empirical), explained by hidden causal powers and mechanisms operating at deeper layers of reality that result in events but which may or may not be overtly observable (in contrast with positivism) and can exist separate to people’s experiences or knowledge of them (in contrast with pragmatism).^17^ CR is methodologically pluralist, but typically privileges intensive, qualitative work as best suited to identifying generative mechanisms in context.^18^ Quantitative methods are nevertheless valued, primarily as extensive^18^ work detecting patterns across populations rather than explaining why those patterns occur. CR uses theory iteratively and critically, through an analytic process called* retroduction*.^17^ Retroduction overlaps considerably with pragmatic abduction but differs because theory is used early and throughout, where competing theories are used to provide explanatory depth about the forces behind lived experience events.^19^ CR suffers from being constrained by an exacting, highly specialised, vocabulary that can be rhetorically off-putting and, at times, self-defeating when it obscures insights for those outside a small circle of specialists.^19^

## Illustrations: Climate and Populism

 Here I provide hypothetical examples of how the four approaches can develop knowledge and understanding about climate change and populism for public health policy – specifically focused on increased heat events and populist mobilization against health directives. I situate CR as a connecting approach across each.

###  Climate Change: From Measurement to Action and Reform

 A typical positivist research question is: *What is the effect of heatwave days above 35 °C on emergency department admissions?* Methods and metrics would rely on time-series analysis and quasi-experimental designs focussed on admissions avoided and mortality reduction. Policy recommendations include heat alerts, surge staffing, and cooling centres. Limitations include ignoring social context such as housing and energy poverty. CR adds value by explaining how these quantitative effects reflect the activation of deeper mechanisms and what policy levers can shift action: eg, rental standards determining cooling options; energy tariffs shaping affordability of green infrastructure especially for lower incomes; labour regulations protecting the exposure of outdoor workers.

 A pragmatic research question asks: *Which local heatwave response practices reduce emergency department wait times?* Methods and metrics combine mixed methods and iterative testing of different bundles of heatwave alerts, adjusted rostering, improvements to local cooling infrastructure spaces and transport access, wait times and safety indicators. Policy recommendations emphasize co-designed interventions and tailored adaptive rollout at scale. Limitations include neglecting upstream determinants for context specificity, limiting scalability and impact. CR adds value by identifying enabling conditions across contexts (eg, governance accountability for local and regional place based interventions, community empowerment, and macro service and resource incentives for local innovation).

 For climate change, a CSS research question would ask: *How do housing, energy, and labour policies create heat vulnerability?* Methods and metrics involve multi-level analysis and institutional tracing about the setting of policies, mapping decisions, rules and mandates, and how these impact risks and exposures including proportion of rental properties meeting standards, energy affordability, safe work compliance, and geographic spread of heat vulnerability over time. Policy recommendations target structural reforms in housing, energy pricing, labour protections, and social support. Limitations include avoidance of policy complexity and slow implementation. CR adds value by specifying causal mechanisms (eg, landlord incentives, energy subsidies for low income areas, resourcing, and training enforcement regimes) and how changing rules reconfigures these to reduce health risks. Together, CSS and CR provide a fuller, deeper, case for reform to reduce demonstrated causal harms.

###  Populism: From Compliance Rates to Institutional Trust Mechanisms

 For populism, a research question positivism asks might be: *How does exposure to anti-elite narratives influence compliance with health directives? *Methods and metrics would be population surveys, content analysis of media sources, and compliance and trust indices. Policy Recommendations concern counter-messaging and media literacy. Limitations are oversimplification or avoidance of complex social factors and conditions. CR adds value by explaining how observed associations necessarily are conditioned by trust in institutions and alienation, mediated through increased social disadvantage as well as access to distorting media messaging.

 A pragmatic research question might be: *Which engagement interventions increase trust in public health policy locally?* Mixed methods combine surveys and interviews, rapid feedback loops about the effectiveness of specific engagement activities like citizen juries, participatory budgeting, and transparency dashboards. Metrics would be trust score changes, participation rates and intervention uptake and quality. Policy recommendations would concern embedding trust-building into routine public health outreach. Limitations are risks of tokenistic engagement and limited theoretical depth to explain why interventions work or not given current social and political conditions. CR adds value by explaining how interventions work by activating recognition, empowerment, and accountability under conditions that mix transparency about the risks, authority about the evidence, and engagement as the most important process.

 For populism, a CSS research question might be: *Which macro-level mechanisms and institutionalised practices drive populist mobilization for or against health directives? *Evidence and metrics would be discourse analysis of populist messaging and policy process tracing of directives and counter messaging, representation indices for different groups in policy processes, media plurality scores, and trust trends. Policy recommendations would include transparency mandates and targeted media regulation focussed on counter messaging. Limitations are that findings and recommendations are politically sensitive and slow to implement. CR adds value by explaining how media concentration and economic insecurity combine to condition mobilisation dynamics.

## Conclusion

 This viewpoint has distinguished four fundamental approaches for building evidence and knowledge for and about public health policy. Each approach contributes something indispensable: positivism measures and predicts; pragmatism enables action and adaptation; CSS explains structures and power; and CR provides the connections across each. But each is limited, requiring considered complementarity as opposed to rejection based on a value position about which is correct or valuable and which is not. Box 1 provides a practical guide for considering the value of each in planning, commissioning and reviewing.

**Box 1. Practical Reading Guide for Researchers, Commissioners, and Reviewers**

- When you see strong patterns but thin explanation, add intensive work to identify mechanisms (CR/CSS).
- When you see rich context but limited portability, specify enabling conditions, leverage points, and policy rules (CR).
- When you see rigorous local optimisation, test for equity impacts and interaction with macro drivers (pragmatism + CSS).
- When you see structural critique, require programme-theory translation and feasible intervention points (CSS + CR).
- When you see technical measurement, ask what upstream mechanisms the numbers point to and which levers can move them (positivism + CR).

--------------

Abbreviations: CSS, critical social science; CR, critical realism.

The paper was borne from frustration about lack of acceptance of methodological pluralism within public health policy research and practice. More broadly, a major challenge facing knowledge for health policy remains institutional. The stark reality facing the planet is an unhabitable climate, increasing inequality, an inexorably rise in populist politics fuelled by the distrust of institutions. Despite the evident need for methodological pluralism, funding and institutional incentives still privilege positivist (and to a lesser extent pragmatic) work. This shapes what is studied and taken up, favouring measurable, near-term problems and undervaluing structural, critical, social scientific analysis.^20,21^

 Health policy, and especially public health policy research, is a relatively small but nevertheless vital part of the system that produces knowledge about the world for the betterment of society. Rebalancing is required in the commissioning, funding, and reviewing of our approaches to knowledge if we are to meet the realities of climate emergency, populism, inequity and the political conditions that create and sustain these risks to health.

## Acknowledgements

 Thanks to Dr. Ed Jegasothy for his review of a late draft of this paper.

## Disclosure of artificial intelligence (AI) use

 I used Copilot to help edit the paper and to clarify the arguments and their presentation.

## Ethical issues

 Not applicable.

## Conflicts of interest

 Author declares that he has no conflicts of interest.

## Endnotes


^[1]^ I use the term “approaches.” Positivism and pragmatism are commonly treated in applied research as paradigms that guide assumptions about reality and knowledge. CSS is a critical theoretical orientation rather than a unified paradigm. CR is a metatheoretical/meta methodological framework. See Crotty^3^ for a comprehensive overview.
